# Multifocality in Testicular Cancer: Clinicopathological Correlations and Prognostic Implications

**DOI:** 10.3390/life14020257

**Published:** 2024-02-16

**Authors:** Uros Bumbasirevic, Milos Petrovic, Milica Zekovic, Vesna Coric, Bogomir Milojevic, Nikola Lisicic, David Obucina, Nenad Vasilic, Petar Bulat, Marko Zivkovic, Milica Cekerevac, Nebojsa Bojanic, Aleksandar Janicic

**Affiliations:** 1 Clinic of Urology, University Clinical Center of Serbia, 11000 Belgrade, Serbia; milospet93@gmail.com (M.P.); em2bogomir@yahoo.com (B.M.); nikola.lisicic1@hotmail.com (N.L.); davidobucina@gmail.com (D.O.); drnenadvasilic@gmail.com (N.V.); bulat.petar@yahoo.com (P.B.); markoziv91@gmail.com (M.Z.); bojanicnebojsa@gmail.com (N.B.); 2 Faculty of Medicine, University of Belgrade, 11000 Belgrade, Serbia; 3 Centre of Research Excellence in Nutrition and Metabolism, Institute for Medical Research, National Institute of Republic of Serbia, University of Belgrade, 11000 Belgrade, Serbia; zekovicmilica@gmail.com; 4 Institute of Medical and Clinical Biochemistry, Faculty of Medicine, University of Belgrade, 11000 Belgrade, Serbia; drcoricvesna@gmail.com; 5 Center of Excellence for Redox Medicine, 11000 Belgrade, Serbia; 6 Department of Pathology, University Clinical Centre of Serbia, 11000 Belgrade, Serbia; milicach@yahoo.com

**Keywords:** testicular cancer, testicular GCT, multifocality, prognostic factors

## Abstract

There are limited data regarding the significance of multifocality in testicular cancer patients. This study evaluated the relationship between multifocality and clinicopathological features determined at the time of radical orchiectomy. The study involved 280 consecutive patients who underwent radical orchiectomy between 2018 and 2023. Multifocality was defined as a distinct tumor focus characterized by a group of malignant cells > 1 mm, clearly differentiated from the primary tumor mass. Uni- and multivariate logistic regression analyses were employed to investigate the association between multifocality and histopathological parameters along with potential risk factors for clinical stages II + III. Multifocality was identified in 44 (15.7%) patients. Significantly smaller primary tumors were observed in subjects with multifocality (20.0 mm vs. 30.0 mm, *p* = 0.0001), while those exhibiting monofocality presented a markedly elevated rate of tumors exceeding 4 cm (40.3% vs. 18.2%, *p* = 0.005). Furthermore, multifocality was associated with a significantly higher rate of primary tumors < 2 cm (52.3% vs. 29.2%, *p* = 0.003). Univariate logistic regression analysis revealed a substantial decrease in the likelihood of multifocality occurrence in seminoma patients with tumors > 4 cm (OR = 0.38, *p* = 0.017). Meanwhile, in multivariate logistic regression, multifocality did not emerge as a significant risk factor for clinical stages II + III in either seminoma (*p* = 0.381) or non-seminoma (*p* = 0.672) cases. Our study suggests that multifocality holds no substantial prognostic relevance for clinically advanced disease in testicular cancer patients. The findings indicate that multifocality is associated with smaller primary tumors, particularly those measuring less than 2 cm.

## 1. Introduction

Testicular cancer (TC) is the most prevalent solid malignant tumor in males between the ages of 15 and 40, although accounting for only 1% of all neoplasms in adult males and 5% of all urological malignancies [1]. With an estimated incidence rate ranging from 3 to 10 cases per 100,000 males annually, there has been a notable rise in the incidence of testicular cancer over the past three decades [1,2]. Germ cell tumors (GCTs) are the most prevalent histologic type of testicular cancer, accounting for 90–95% of cases. Seminomas constitute around 55–60% of all GCTs, leaving the remaining 40–45% as non-seminomatous germ cell tumors (NSGCTs) [3]. The incidence of bilateral GCTs ranges from 1% to 5%, with approximately one-third identified as synchronous cases, while the majority are diagnosed as metachronous tumors. [4].

There are several well-established prognostic factors in GCTs. Serum tumor markers, such as beta human chorionic gonadotropin (bHCG), alpha fetoprotein (AFP), and lactate dehydrogenase (LDH), hold acknowledged prognostic significance and are routinely and systematically incorporated into the conventional clinical protocols for the management of testicular cancer [5]. A tumor size > 4 cm and stromal rete testis invasion have been identified as feasible risk factors for the stratification of seminoma clinical stage I patients into low-risk and high-risk groups and determining the need for adjuvant treatment [6]. Similarly, lymphovascular invasion (LVI) has been recognized as a reliable prognostic factor for relapse in clinical stage I NSGCT patients, guiding risk-adapted treatment strategies [7]. Systemic inflammation indices, particularly the neutrophile-to-lymphocyte ratio (NLR), systemic immune-inflammation index (SII) and platelet-to-lymphocyte ratio (PLR), have demonstrated the potential for complementing and improving existing biomarkers and clinicopathological variables in GCT patients [8]. The utilization of advanced techniques like next-generation sequencing (NGS) and multiomics has led to the identification of several promising prognostic factors in TC patients [9,10]. However, the implementation of these prognosticators in clinical practice requires extensive validation through meticulously designed and rigorously conducted studies.

Multifocality denotes the existence of multiple lesions originating monoclonally within a single tissue. This pathological entity has been recognized as an important prognostic factor in several malignancies. The presence of multifocality in breast cancer might potentially impact the management of the primary tumor and influence the decision regarding the dissection of axillary lymph nodes [11]. Several studies have suggested that the presence of multifocality can be associated with an unfavorable prognosis in breast cancer patients [12]. The occurrence of multifocality in thyroid cancer is a significant prognosticator of disease progression and increased risk of disease recurrence [13]. Multifocality is a common phenomenon in prostate cancer as well. A substantial body of research has demonstrated that the majority, namely over 80%, of primary prostate cancers exhibit the presence of multiple distinct tumor foci [14]. The presence of multifocality holds the potential to exert a substantial impact on the strategic considerations for focal therapy in this particular patient cohort [15].

The relevance and clinical implications of multifocality in testicular cancer have received limited research attention, as evidenced by the scarcity of publications investigating this phenomenon [16,17,18]. While radical orchiectomy still presents a standard treatment for suspicious testicular lesions, there is a valid argument for considering testis-sparing surgery (TSS) in some clinical scenarios, such as synchronous bilateral tumors or tumors affecting a solitary testis [19]. Furthermore, testis-sparing surgery can be offered to patients with small, indeterminate testicular lesions, a normal contralateral testicle, and negative tumor markers, with the aim to preserve gonadal function [20]. Therefore, the possible implications of multifocality in individuals who may be suitable candidates for testis-sparing surgery are of considerable importance.

Given the aforementioned implications and the requirement for new prognostic markers in TC patients, our study’s objective was to ascertain the relationship between multifocality and other clinical and pathological variables in testicular GCT patients at the time of radical orchiectomy.

## 2. Materials and Methods

### 2.1. Patients and Preoperative Assessment

This study involved a cohort of 280 consecutive patients who underwent radical orchiectomy at the Clinic of Urology, University Clinical Centre of Serbia, Belgrade, over the period ranging from January 2018 to August 2023. A detailed preoperative assessment was conducted in all study participants. After acquiring a comprehensive clinical history, a thorough physical examination was conducted, subsequently followed by scrotal ultrasonography to determine the dimensions and location of the testicular tumor. Computerized tomography (CT) of the chest, abdomen and pelvis was conducted to evaluate the potential existence of metastases. Alternatively, a CT scan of the abdomen and pelvis, coupled with chest radiography, was employed for the same purpose. The laboratory examinations encompassed a complete blood count (CBC) and biochemical analysis, which involved the assessment of serum tumor markers including LDH, bHCG and AFP. The tumor markers were reanalyzed at least one week post-surgery.

### 2.2. Clinical Staging and Histopathological Evaluation

The staging was performed in accordance with the tumor, node and metastasis (TNM) classification system, as outlined in the 8th edition of the Union for International Cancer Control (UICC) from 2016 [21]. The clinical stage was defined as stage I if the tumor was limited to the testis, stage II if there were regional retroperitoneal lymph node metastases and stage III if distant metastases beyond the regional retroperitoneal nodes were detected. The International Germ Cell Cancer Collaborative Group (IGCCCG) criteria were employed to conduct risk stratification for patients who presented with metastatic disease [22]. We study classified patients into two separate cohorts: (a) patients presented with localized disease (clinical stage I), and (b) patients presented with advanced disease (clinical stages II and III).

The orchiectomy specimens underwent histopathological evaluation by a dedicated pathological team with extensive expertise in uro-oncology, operating within the largest healthcare facility in the country. Detected testicular GCTs were classified in accordance with the World Health Organization (WHO) classification [23,24]. The pathological characteristics evaluated for each orchiectomy specimen encompassed the assessment of primary (index) tumor mass dimensions, identification of multiple tumor foci, examination of tumor cell infiltration into the vascular vessels, lymphatic vessels and the rete testis, and detection of germ cell neoplasia in situ (GCNis). The term “multifocality” was defined as the presence of a distinct tumor focus characterized by a group of malignant cells measuring greater than 1 mm, which can be clearly differentiated from the primary tumor mass.

### 2.3. Ethical Considerations

The study protocol received approval from the Ethics Board of the University Clinical Centre of Serbia, with reference number 717/9, and the Ethics Board of the Faculty of Medicine, University of Belgrade, with reference number 1322/IX-15. Written informed consent was obtained from all participants involved in this study. All research endeavors adhered rigorously to the ethical guidelines and principles set forth by these esteemed institutions. This study also conformed to the ethical standards articulated in the Declaration of Helsinki.

### 2.4. Statistical Analysis

The statistical analysis was performed using the Statistical Package for Social Sciences 22.0 (SPSS Inc., Chicago, IL, USA). The Mann–Whitney U test was employed to examine the continuous variables, and the chi-squared test or Fisher’s exact test, depending on appropriateness, was utilized to conduct statistical analysis on the qualitative data. The odds ratios between multifocality and various histopathological parameters were analyzed using univariate logistic regression. Multivariate logistic regression analysis was employed in order to detect potential risk factors for the occurrence of clinically advanced disease (clinical stages II + III). All *p*-values were calculated with a two-sided test, with *p* < 0.05 considered statistically significant.

## 3. Results

Baseline clinical and histological characteristics are displayed in Table 1 and Table 2. The median age of patients at diagnosis was 33.4 years (range 17–57). A total of 146 patients (52.1%) were diagnosed with left-sided tumors, whereas 134 patients (47.9%) had right-sided tumors. The majority of patients (72.5%) were diagnosed in clinical stage I, with clinical stages II and III being assigned to 17.5% and 10% of patients, respectively. The vast majority of patients (72.5%) presented with non-metastatic disease. Regarding the IGCCCG prognostic risk groups for metastatic GCTs, 46 out of a total of 280 patients had a good prognosis (16.4%), with 13 patients (4.6%) each in both the intermediate and poor prognosis risk groups.

Out of the total number of 280 patients, 155 (55.4%) were diagnosed with pure seminoma, whereas 125 (44.6%) had a non-seminomatous histology, which also included mixed testicular GCTs with a seminoma component. The most prevalent histology among NSGCTs was embryonal carcinoma, accounting for 34.6% of cases. A yolk sac tumor was the second most frequent, representing 26.8% of cases, followed by teratoma at 25.4%. Choriocarcinoma was the least common histology, with a prevalence of 11.1%. Forty-four orchiectomy specimens (15.7% of patients) exhibited multifocality.

**Figure 1:**
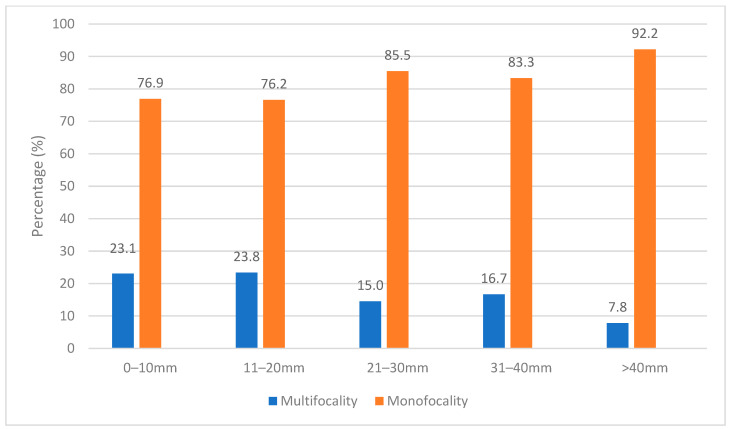
Percentage of multifocality (blue) and monofocality (orange) in regard to specific index tumor size; SD—standard deviation.

The association between multifocality and various clinicopathological parameters is exhibited in Table 3 and Figure 1. In patients with multifocality, the median size of the primary tumor mass was 20 mm, ranging from 6 to 70 mm. Conversely, in patients with monofocality, the median tumor size was 30 mm (range 3 to 125). Compared to patients with monofocal tumors, patients with multifocality had significantly smaller index tumors (*p* = 0.001). The rate of tumor size > 4 cm was statistically significantly higher in patients with monofocality compared to patients with multifocality (40.3% vs. 18.2%, *p* = 0.005). Additionally, multifocality was associated with a significantly higher rate of index tumors < 2 cm in comparison to monofocal cases (52.3% vs. 29.2%, *p* = 0.003).

Multifocality was detected in 18.1% of patients with a pure seminoma histology and 12.8% of NSGCT patients. No statistically significant connection between multifocality and histology was observed (*p* = 0.150). In relation to the clinical stage of disease, multifocality was diagnosed in 15.8% of CS I patients, 16.3% of CS II patients and 14.3% of CS III patients. There was no statistically significant association between multifocality and the clinical stage of disease (*p* = 0.972). In addition, there was no significant difference detected in the incidence of multifocality between patients with clinically localized (CS I) and advanced (CS II + III) disease (15.8% vs. 15.6%, *p* = 0.971). Furthermore, no statistical significance was identified in the relationship between multifocality rate and pathological stage (*p* = 0.218).

In patients with seminoma, univariate logistic regression analysis showed a significant association between multifocality and the size of the primary tumor < 4 cm (*p* = 0.017) (Table 4). In the same analysis, rete testis was not linked with multifocality (*p* = 0.667). Regarding non-seminomatous histology, an association between multifocality and lymphovascular invasion (*p* = 0.463), the presence of embryonal carcinoma (*p* = 0.179), choriocarcinoma (*p* = 0.226), yolk sac tumor (*p* = 0.427) and teratoma (*p* = 0.983) was not found.

The multivariate logistic regression analysis was employed to identify potential pathological risk factors of clinically advanced disease development (Table 5). For patients with a pure seminoma histology, variables included in the model were a tumor size > 4 cm, rete testis invasion and multifocality. Among these variables, only a tumor size > 4 cm was a significant risk factor of clinically advanced disease (OR = 3.54, 95% CI: 1.22–10.29, *p* = 0.02). Although rete testis invasion and multifocality were associated with an increased risk of a clinically advanced stage of disease, statistical significance was not established. In relation to the NSGCT histology, variables included in the model for multivariate logistic regression analysis were LVI and multifocality. No statistically significant risk factors were identified for the development of clinically advanced disease.

## 4. Discussion

The importance of multiple pathological prognostic factors in the process of decision-making and selection of an appropriate treatment strategy is well-established in patients with testicular cancer [25]. Following preliminary research that indicated primary tumor size as a possible prognosticator of disease relapse in CSI seminoma patients under active surveillance, a pooled analysis from 2002 validated these findings while also detecting rete testis invasion as an additional prognostic factor of relapse [26,27,28,29,30]. In this study, the tumor size > 4 cm and rete testis invasion were found to be significant and independent prognostic factors, with hazard ratios of 2.0 and 1.7, respectively, while the presence of both variables was associated with a hazard ratio of 3.4 [28]. In light of the previously outlined trials, it is suggested that patients exhibiting these prognostic factors would derive benefits from adjuvant treatment. Currently, a tumor size > 4 cm and rete testis invasion are implemented as risk factors for the stratification of CSI seminoma patients into low-risk and high-risk groups and for determining the need for adjuvant treatment [6,29,30]. Nevertheless, these prognostic indicators have been shown to have substantial limitations. Studies have found a linear correlation between the size of the primary tumor and the probability of disease relapse, thus indicating that there is no clear basis for using a specific threshold value [31]. Furthermore, there is a high prevalence, reaching up to 50% of cases, of misrecognition and the failure to discern invasion of the rete testis [31]. Such considerations may have considerable ramifications for the utilization of these pathological parameters in standard clinical practice.

The prognostic significance of various pathological variables was also investigated in patients with the NSGCT histology. Several studies have found the proportion of embryonal carcinoma, proliferation rate and LVI to be possible prognostic factors of disease recurrence in CSI NSGCT patients [32,33]. LVI, in the context of multivariate analysis, supersedes other risk factors and is hence employed to categorize stage I NSGCT patients into distinct “high-risk” and “low-risk” categories [29,30,34]. The risk-adapted treatment strategy relies on the discerning capabilities of LVI, where patients designated as LVI+ (i.e., “high-risk”) exhibit a recurrence rate of up to 50%, contrasting with LVI− (i.e., “low-risk”) patients who demonstrate a lower recurrence rate of merely 14%. This distinction informs the recommendation of risk-adapted treatment, entailing adjuvant therapy for high-risk patients and active surveillance for those deemed low-risk, while considering individual patient preferences as well [25,29].

Given the aforementioned constraints of current prognostic factors and the need for supplementary pathological indicators in patients with TC, we examined the prevalence and prognostic importance of multifocality in radical orchiectomy specimens.

The finding of multifocality has well-established significance in several solid malignant tumors. Numerous tumors have the capacity to manifest as multiple lesions within a tissue, a phenomenon whose origin—whether independent or monoclonal—has sparked considerable debate regarding its relevance to tumor staging, progression and the selection of appropriate therapeutic strategies. Sophisticated mathematical models have revealed that the genesis of multifocal cancers is intricately linked to the dynamic interplay between localized tumor-promoting and longer-range tumor-inhibiting factors. Such a paradigm posits that multifocal cancers represent an intermediary phase in cancer progression, signifying a shift from inhibition toward promotion. Notably, distinct progression patterns unfold: under robust tumor inhibition, the initial phase witnesses unifocal growth, succeeded by bifurcation and the inception of multiple lesions. As the tumor undergoes evolution, tipping the equilibrium toward promotion, these lesions eventually coalesce into a singular, formidable mass with the capability of tissue invasion. Conversely, a more subdued tumor inhibition at initiation facilitates solitary lesion growth until the entire tissue succumbs to invasion [35].

The presence of multiple tumor foci in breast cancer is generally regarded as a contraindication for conservative surgery given the increased risk of local recurrence [11]. While also occurring more commonly in younger patient populations, multifocal breast cancer is characterized by aggressive behavior, a higher proclivity for metastatic dissemination and lymph node involvement [36]. Furthermore, a 2014 meta-analysis demonstrated an association between multifocality and worse overall survival in patients with breast cancer [12]. In thyroid cancer, multifocality is a significant indicator of disease progression and an increased probability of recurrence [13].

To the best of our knowledge, only three studies have assessed the predictive importance of multifocality in TC and its association with other histopathological factors [16,17,18], and we present the largest series in the literature. In a study from 2009, Ehrlich and colleagues evaluated multifocality in radical orchiectomy specimens from 145 consecutive patients [16]. Multifocality was broadly defined as 1 of 4 possible distinct pathological entities, including “distinct tumor focus > 1 mm that is separable from the main tumor mass, microinvasive tumor characterized by a single or small group of malignant germ cells scattered within the normal interstitial parenchyma, extra tumor vascular invasion, and rete testis invasion by pagetoid tumor spread”. According to this definition, multifocality was detected in 33% of patients and was statistically significantly more common in seminoma patients (*p* = 0.007). The presence of multifocality was not of prognostic significance, as it was not related with either an advanced clinical stage of disease (CSII + III, *p* = 0.333) or intermediate + poor IGCCCG prognostic risk group (*p* = 0.989) [16]. In a study from 2014 that included 254 consecutive patients who underwent radical orchiectomy, Favilla et al. defined multifocality as “the presence of a distinct tumor focus of cluster of malignant cells greater than 0.5 mm and conspicuously separable from the main tumor mass” [17]. A total of 22.83% patients had multifocality. Contrary to the study of Ehrlich et al., statistical analysis did not reveal any association between tumor histological characterization and multifocality (*p* = 0.95). In patients with seminomatous histology, a univariate logistic regression analysis failed to detect an association between multifocality and other well-established pathological features, such as tumor size more than 4 cm and rete testis invasion (*p* = 0.72 and *p* = 0.25, respectively). Likewise, no substantial association was identified between LVI or the proportion of embryonal carcinoma exceeding 50% and multifocality (*p* = 0.09 and *p* = 0.99, respectively). In the multivariate logistic regression analysis, multifocality was not shown to be a negative pathological risk factor for clinically advanced disease development (*p* = 0.23) or pathological stage (*p* = 0.30) [17]. In a subsequent 2015 study conducted by the same group of authors, which focused on the presence of multifocality and testicular intraepithelial neoplasia (TIN) in patients eligible for testis-sparing surgery, the findings echoed the previously reported results as multifocality did not demonstrate an association with tumor histology or advanced clinical and pathological stages [18].

In our study, we adopted a more stringent criterion for multifocality. Specifically, we defined it as the presence of a distinct tumor focus characterized by a group of malignant cells measuring greater than 1 mm, which can be clearly differentiated from the primary tumor mass. Consequently, multifocality was detected in 15.7% of patients. This rate was markedly lower than the figures reported by Favila et al. in 2014 and 2015 (22.83% and 26.19%, respectively), and notably less than the multifocality rate of 33% documented in Ehrlich’s 2009 study. The considerable variance in multifocality rates, particularly in comparison to Ehrlich’s study can be attributed to significantly different definitions of multifocality. Ehrlich implemented a broader definition, encompassing four distinct pathological entities, potentially leading to a significant overestimation of multifocality prevalence. Our investigation demonstrated a non-significant discrepancy in multifocality rates between seminoma and NSCGT patients, in line with the observations reported by Favilla et al. in 2014. [17]. Furthermore, we utilized univariate logistic regression analysis to determine the possible association between multifocality and other pathological features. Contrasting with previously published results, our study revealed a significant link between multifocality and tumor size > 4 cm in the seminoma patient group (OR = 0.38, *p* = 0.017), thereby indicating a reduced likelihood of multifocality in tumors larger than 4 cm. In NSGCT patients, LVI was not associated with the presence of multifocality (*p* = 0.463). Diverging from earlier investigations, we conducted an additional analysis to examine the relationship between multifocality and the occurrence of embryonal cancer, choriocarcinoma, yolk sac tumor, or teratoma, but no significant relationship was discerned. As previously stated, tumor size > 4 cm, RTI, and LVI are well-established pathological variables that are linked to an increased risk of advanced clinical stage at the time of diagnosis in patients with TC [26,27,28,29,30]. To assess the predictive significance of multifocality in relation to the higher likelihood of clinically advanced disease, we included this pathological parameter in a multivariate logistic regression analysis, along with additional established pathological features. Consistent with the reported findings of Ehrlich in 2009 and Favilla in 2014 [16,17], our model revealed that multifocality did not emerge as a significant prognostic factor for clinically advanced disease development.

The finding of multifocality can have significant implications for patients who are eligible for organ-sparing surgery. According to the current guidelines, partial orchiectomy (PO) is considered a valid treatment option in a setting of a congenitally acquired or functionally solitary testis or bilateral synchronous tumors [25,37]. Based on the American Urological Association (AUA) guideline, PO can be offered as an alternative to radical orchiectomy in carefully selected patients wishing to preserve hormonal and fertility function with masses < 2 cm, equivocal physical exam or ultrasonography findings and negative tumor markers [37]. There is a strong rationale for utilization of PO in a patients with non-palpable testicular masses < 2 cm. It is estimated that around 50–80% of non-palpable testicular masses measuring less than 2 cm are benign lesions, including testicular cysts, minor infarcts or sex cord stromal tumors [20,38,39]. According to a meta-analysis conducted in 2020, the local recurrence rate after TSS was 10.9% [40]. Local recurrences were observed in 20.3% of patients who did not receive systemic chemotherapy or local radiation to the ipsilateral testicle as adjuvant treatment [40]. The presence of multifocality might have significant long-term implications for the oncological safety of TSS, potentially leading to an alteration in treatment approach for suitable patients.

Our study found that patients with multifocality had a significantly smaller main tumor mass compared to patients with monofocal tumors (20 mm vs. 30 mm, *p* = 0.0001). Further analysis indicated that tumors larger than 4 cm were substantially more frequent in patients with monofocal tumors. Additionally, a univariate logistic regression analysis concurred that seminoma patients with tumors greater than 4 cm had a considerably reduced probability of exhibiting multifocality. A total of 92 patients in our cohort had a tumor with a diameter of 2 cm or less. Within this group of patients, 25% had multifocality. In contrast to the study conducted by Ehrlich and colleagues, in which they reported a multifocality rate of 63% among patients with tumors smaller than 2 cm [16], we observed a significantly lower multifocality rate. This discrepancy can be attributed to the previously stated difference in the criteria of multifocality. Nevertheless, our multifocality rate corresponds more closely with the observed local recurrence rate following TSS, standing at 20.3% or patients without adjuvant treatment [40]. Subsequent analysis revealed that multifocality was associated with a significantly higher rate of index tumors < 2 cm in comparison to monofocality (52.3% vs. 29.2%, *p* = 0.003). Overall, our study’s findings substantiate the previously suggested hypothesis that smaller index tumors are more frequently correlated with multifocality due to reduced compression on the surrounding uninvolved parenchyma, thus facilitating the identification of other tumor foci [16]. The results of our study indicate that the presence of multiple foci is linked to smaller primary tumors, particularly those measuring less than 2 cm. Urologists should be aware of this association as it may pose a risk to oncological safety when considering a testis-sparing approach in these patients.

However, our study is subject to several inherent limitations that warrant careful consideration. Primarily, the retrospective design of this study introduces a contextual and methodological framework that should be acknowledged when interpreting the results. Secondly, the lack of follow-up data restricted the assessment of the relationship between multifocality and the likelihood of relapse. Furthermore, it is imperative to acknowledge the inherent variability in pathology interpretation, which can significantly impact study outcomes. The intricate nature of the testicular pathology and the relative rarity of this uro-oncoclogical entity pose formidable challenges to the effective evaluation of orchiectomy specimens [41,42]. It is crucial to note, however, that the histopathological evaluation in our study was centralized, and it was conducted by a dedicated pathological team with extensive expertise in uro-oncology, operating within the largest healthcare facility in the country, thus leveraging the clinic’s pivotal role in providing specialized medical care. Finally, our patient group consisted of consecutive, unselected individuals who underwent radical orchiectomy for TGCTs. Hence, the findings of our study cannot be fully extrapolated to carefully selected patients who are suitable candidates for TSS. Nevertheless, the study’s strength lies in its ability to provide invaluable insights into a topic characterized by insufficient data in the existing literature. Notably, our study boasts the distinction of presenting the largest patient cohort to date within this field, coupled with a comprehensive central pathological review.

## 5. Conclusions

The results of our study indicate that multifocality should not be regarded as a significant prognostic factor of clinically advanced disease in patients with TC at initial presentation. Our data also suggest that the presence of multifocality is associated with smaller primary tumors, particularly those measuring less than 2 cm.

## Figures and Tables

**Table 1:** Baseline clinical characteristics.

Variable	
Age (years), mean ± SD	33.42 ± 8.13
Side, n (%)	
Right	134 (47.9)
Left	146 (52.1)
Tumor size (mm), median (IQR)	35.38 (3–125)
Clinical stage, n (%)	
I	203 (72.5)
II	49 (17.5)
III	28 (10)
IGCCCG risk group, n (%)	
Non-metastatic disease	208 (74.3)
Poor prognosis	46 (16.4)
Intermediate prognosis	13 (4.6)
Poor prognosis	13 (4.6)

SD—standard deviation; IQR—interquartile range; IGCCCG—International Germ Cell Cancer Collaborative Group.

**Table 2:** Histological characteristics.

Variable	
Histology, n (%)	
Seminoma	155 (55.4)
NSGCT	125 (44.6)
Pathological stage, n (%)	
pT1	126 (45.0)
pT2	133 (47.5)
pT3	19 (6.8)
pT4	2 (0.7)
Multifocality, n (%)	
Yes	44 (15.7)
No	236 (84.3)

NSGCT—non-seminomatous testicular germ cell tumors.

**Table 3:** Association between multifocality, monofocality and clinicopathological parameters.

Variable	Multifocality	Monofocality	*p*-Value
Tumor size (mm), median	20 (6–7)	30 (3–125)	0.001
Tumor size > 4 cm, n (%)			0.005
Yes	8 (18.2)	95 (40.3)	
No	36 (81.8)	141 (59.7)	
Tumor size < 2 cm, n (%)			0.003
Yes	23 (52.3)	69 (29.2)	
No	21 (47.7)	167 (70.8)	
Histologic type, n (%)			0.150
Seminoma	28 (18.1)	127 (81.9)	
NSGCT	16 (12.8)	109 (87.2)	
Clinical stage, n (%)			0.972
I	32 (15.8)	171 (84.2)	
II	8 (16.3)	41 (83.7)	
III	4 (14.3)	24 (85.7)	
Clinical stage, n (%)			0.971
Localized disease (CS I)	32 (15.8)	171 (84.2)	
Advanced disease (CSII + III)	12 (15.6)	65 (84.4)	
Pathological stage			0.218
pT1	24 (19.0)	102 (81.0)	
pT2	18 (13.5)	115 (86.5)	
pT3	1 (5.3)	18 (94.7)	
pT4	1 (50)	1 (50)	

CS—clinical stage.

**Table 4:** The risk of multifocality in relation to the presence of specific clinicopathological characteristics.

Variable	OR (95% CI)	*p*-Value
Seminoma		
Tumor size (>4 cm vs. <4 cm)	0.38 (0.29–0.46)	0.017
Rete testis invasion	0.40 (0.32–0.49)	0.667
NSCGT		
LVI	0.72 (0.63–0.81)	0.463
Embryonal carcinoma	0.78 (0.69–0.86)	0.179
Choriocarcinoma	0.27 (0.18–0.35)	0.226
Yolk sac	0.61 (0.51–0.69)	0.427
Teratoma	0.56 (0.46–0.65	0.983

NSGCT—non-seminomatous germ cell tumor; OR—odds ratio; CI-confidence interval; LVI—lymphovascular invasion.

**Table 5:** The risk of clinically advanced disease development in relation to the presence of specific clinicopathological features.

Variable	OR (95% CI)	*p*-Value
Seminoma		
Tumor size > 4 cm	3.54 (1.22–10.29)	0.020
Rete testis invasion	1.41 (0.51–3.93)	0.507
Multifocality	1.76 (0.49–6.26)	0.381
NSCGT		
LVI		0.099
Multifocality	1.96 (0.89–4.34) 1.26 (0.43–3.66)	0.672

OR—odds ratio; CI—confidence interval; NSCGT—non-seminomatous germ cell tumor; LVI—lymphovascular invasion.

## Data Availability

The data supporting the reported results can be found upon request in the form of datasets available at the Clinic of Urology, University Clinical Centre of Serbia and at the LymeSurvey repository, https://upitnik.med.bg.ac.rs, Institute of Medical and Clinical Biochemistry, Faculty of Medicine, University of Belgrade.

## References

[1] Park J.S., Kim J., Elghiaty A., Ham W.S. (2018). Recent global trends in testicular cancer incidence and mortality. Medicine.

[2] Gurney J.K., Florio A.A., Znaor A., Ferlay J., Laversanne M., Sarfati D., Bray F., McGlynn K.A. (2019). International Trends in the Incidence of Testicular Cancer: Lessons from 35 Years and 41 Countries. Eur. Urol..

[3] Rajpert-De Meyts E., McGlynn K.A., Okamoto K., Jewett M.A., Bokemeyer C. (2016). Testicular germ cell tumours. Lancet..

[4] Sarıcı H., Telli O., Eroğlu M. (2013). Bilateral testicular germ cell tumors. Turk. J. Urol..

[5] Dieckmann K.P., Simonsen-Richter H., Kulejewski M., Anheuser P., Zecha H., Isbarn H., Pichlmeier U. (2019). Serum Tumour Markers in Testicular Germ Cell Tumours: Frequencies of Elevated Levels and Extents of Marker Elevation Are Significantly Associated with Clinical Parameters and with Response to Treatment. Biomed. Res. Int..

[6] Bumbasirevic U., Zivkovic M., Petrovic M., Coric V., Lisicic N., Bojanic N. (2022). Treatment options in stage I seminoma. Oncol. Res..

[7] Leman E.S., Gonzalgo M.L. (2010). Prognostic features and markers for testicular cancer management. Indian. J. Urol..

[8] Janicic A., Petrovic M., Zekovic M., Vasilic N., Coric V., Milojevic B., Zivkovic M., Bumbasirevic U. (2023). Prognostic Significance of Systemic Inflammation Markers in Testicular and Penile Cancer: A Narrative Review of Current Literature. Life.

[9] Leão R., Albersen M., Looijenga L.H.J., Tandstad T., Kollmannsberger C., Murray M.J., Culine S., Coleman N., Belge G., Hamilton R.J. (2021). Circulating MicroRNAs, the Next-Generation Serum Biomarkers in Testicular Germ Cell Tumours: A Systematic Review. Eur. Urol..

[10] Lobo J., Alzamora M.A., Guimarães R., Cantante M., Lopes P., Braga I., Maurício J., Jerónimo C., Henrique R. (2020). p53 and MDM2 expression in primary and metastatic testicular germ cell tumors: Association with clinical outcome. Andrology..

[11] Bendifallah S., Werkoff G., Borie-Moutafoff C., Antoine M., Chopier J., Gligorov J., Uzan S., Coutant C., Rouzier R. (2010). Multiple synchronous (multifocal and multicentric) breast cancer: Clinical implications. Surg. Oncol..

[12] Vera-Badillo F.E., Napoleone M., Ocana A., Templeton A.J., Seruga B., Al-Mubarak M., AlHashem H., Tannock I.F., Amir E. (2014). Effect of multifocality and multicentricity on outcome in early stage breast cancer: A systematic review and meta-analysis. Breast Cancer Res. Treat..

[13] Joseph K.R., Edirimanne S., Eslick G.D. (2018). Multifocality as a prognostic factor in thyroid cancer: A meta-analysis. Int. J. Surg..

[14] Haffner M.C., Zwart W., Roudier M.P., True L.D., Nelson W.G., Epstein J.I., De Marzo A.M., Nelson P.S., Yegnasubramanian S. (2021). Genomic and phenotypic heterogeneity in prostate cancer. Nat. Rev. Urol..

[15] Jaipuria J., Ahmed H.U. (2022). Clinical and pathologic characteristics to select patients for focal therapy or partial gland ablation of nonmetastatic prostate cancer. Curr. Opin. Urol..

[16] Ehrlich Y., Konichezky M., Yossepowitch O., Baniel J. (2009). Multifocality in testicular germ cell tumors. J. Urol..

[17] Favilla V., Russo G.I., Spitaleri F., Urzì D., Garau M., Madonia M., Saita A., Pirozzi Farina F., La Vignera S., Condorelli R. (2014). Multifocality in testicular germ cell tumor (TGCT): What is the significance of this finding?. Int. Urol. Nephrol..

[18] Favilla V., Russo G.I., Spitaleri F., Urzì D., Madonia M., La Vignera S., Condorelli R., Calogero A.E., Cimino S., Morgia G. (2015). Prevalence of intratubular germ cell neoplasia and multifocality in testicular germ cell tumors ≤ 2 cm: Relationship with other pathological features. Clin. Genitourin. Cancer.

[19] Bojanic N., Bumbasirevic U., Vukovic I., Bojanic G., Milojevic B., Nale D., Durutovic O., Djordjevic D., Nikic P., Vuksanovic A. (2015). Testis sparing surgery in the treatment of bilateral testicular germ cell tumors and solitary testicle tumors: A single institution experience. J. Surg. Oncol..

[20] Bojanic N., Bumbasirevic U., Bojanic G., Vukovic I., Milojevic B., Pekmezovic T. (2017). Testis sparing surgery for treatment of small testicular lesions: Is it feasible even in germ cell tumors?. J. Surg. Oncol..

[21] Brieley J.E., Gospodarowicz M.K., Wittekind C. (2016). The TNM Classification of Malignant Tumours.

[22] Gillessen S., Sauvé N., Collette L., Daugaard G., de Wit R., Albany C., Tryakin A., Fizazi K., Stahl O., Gietema J.A. (2021). International Germ Cell Cancer Classification Update Consortium. Predicting Outcomes in Men With Metastatic Nonseminomatous Germ Cell Tumors (NSGCT): Results From the IGCCCG Update Consortium. J. Clin. Oncol..

[23] Moch H., Cubilla A.L., Humphrey P.A., Reuter V.E., Ulbright T.M. (2016). The 2016 WHO Classification of Tumours of the Urinary System and Male Genital Organs-Part A: Renal, Penile, and Testicular Tumours. Eur. Urol..

[24] Moch H., Amin M.B., Berney D.M., Compérat E.M., Gill A.J., Hartmann A., Menon S., Raspollini M.R., Rubin M.A., Srigley J.R. (2022). The 2022 World Health Organization Classification of Tumours of the Urinary System and Male Genital Organs-Part A: Renal, Penile, and Testicular Tumours. Eur. Urol..

[25] Patrikidou A., Cazzaniga W., Berney D., Boormans J., de Angst I., Di Nardo D., Fankhauser C., Fischer S., Gravina C., Gremmels H. (2023). European Association of Urology Guidelines on Testicular Cancer: 2023 Update. Eur. Urol..

[26] von der Maase H., Specht L., Jacobsen G.K., Jakobsen A., Madsen E.L., Pedersen M., Rørth M., Schultz H. (1993). Surveillance following orchidectomy for stage I seminoma of the testis. Eur. J. Cancer.

[27] Warde P., Gospodarowicz M.K., Banerjee D., Panzarella T., Sugar L., Catton C.N., Sturgeon J.F., Moore M., Jewett M.A. (1997). Prognostic factors for relapse in stage I testicular seminoma treated with surveillance. J. Urol..

[28] Warde P., Specht L., Horwich A., Oliver T., Panzarella T., Gospodarowicz M., von der Maase H. (2002). Prognostic factors for relapse in stage I seminoma managed by surveillance: A pooled analysis. J. Clin. Oncol..

[29] Oldenburg J., Aparicio J., Beyer J., Cohn-Cedermark G., Cullen M., Gilligan T., De Giorgi U., De Santis M., de Wit R., Fosså S.D. (2015). Personalizing, not patronizing: The case for patient autonomy by unbiased presentation of management options in stage I testicular cancer. Ann. Oncol..

[30] Honecker F., Aparicio J., Berney D., Beyer J., Bokemeyer C., Cathomas R., Clarke N., Cohn-Cedermark G., Daugaard G., Dieckmann K.P. (2018). ESMO Consensus Conference on testicular germ cell cancer: Diagnosis, treatment and follow-up. Ann. Oncol..

[31] Chung P., Daugaard G., Tyldesley S., Atenafu E.G., Panzarella T., Kollmannsberger C., Warde P. (2015). Evaluation of a prognostic model for risk of relapse in stage I seminoma surveillance. Cancer Med..

[32] Sturgeon J.F., Moore M.J., Kakiashvili D.M., Duran I., Anson-Cartwright L.C., Berthold D.R., Warde P.R., Gospodarowicz M.K., Alison R.E., Liu J. (2011). Non-risk-adapted surveillance in clinical stage I nonseminomatous germ cell tumors: The Princess Margaret Hospital’s experience. Eur. Urol..

[33] Heidenreich A., Schenkmann N.S., Sesterhenn I.A., Mostofi F.K., McCarthy W.F., Heidenreich B., Moul J.W. (1997). Immunohistochemical expression of Ki-67 to predict lymph node involvement in clinical stage I nonseminomatous germ cell tumors. J. Urol..

[34] Winter C., Hiester A. (2021). Treatment of clinical stage I non-seminoma. Asian J. Urol..

[35] Wodarz D., Iwasa Y., Komarova N.L. (2004). On the emergence of multifocal cancers. J. Carcinog..

[36] Wolters R., Wöckel A., Janni W., Novopashenny I., Ebner F., Kreienberg R., Wischnewsky M., Schwentner L. (2013). BRENDA Study Group. Comparing the outcome between multicentric and multifocal breast cancer: What is the impact on survival, and is there a role for guideline-adherent adjuvant therapy? A retrospective multicenter cohort study of 8935 patients. Breast Cancer Res. Treat..

[37] Stephenson A., Bass E.B., Bixler B.R., Daneshmand S., Kirkby E., Marianes A., Pierorazio P.M., Sharma R., Spiess P.E. (2024). Diagnosis and Treatment of Early-Stage Testicular Cancer: AUA Guideline Amendment 2023. J. Urol..

[38] Müller T., Gozzi C., Akkad T., Pallwein L., Bartsch G., Steiner H. (2006). Management of incidental impalpable intratesticular masses of < or = 5 mm in diameter. BJU Int..

[39] Rolle L., Tamagnone A., Destefanis P., Bosio A., Timpano M., Fiori C., Ceruti C., Burlo P., Fauciglietti P., Fontana D. (2006). Microsurgical “testis-sparing” surgery for nonpalpable hypoechoic testicular lesions. Urology.

[40] Patel H.D., Gupta M., Cheaib J.G., Sharma R., Zhang A., Bass E.B., Pierorazio P.M. (2020). Testis-sparing surgery and scrotal violation for testicular masses suspicious for malignancy: A systematic review and meta-analysis. Urol. Oncol..

[41] Berney D.M., Algaba F., Amin M., Delahunt B., Compérat E., Epstein J.I., Humphrey P., Idrees M., Lopez-Beltran A., Magi-Galluzzi C. (2015). Handling and reporting of orchidectomy specimens with testicular cancer: Areas of consensus and variation among 25 experts and 225 European pathologists. Histopathology.

[42] Harari S.E., Sassoon D.J., Priemer D.S., Jacob J.M., Eble J.N., Caliò A., Grignon D.J., Idrees M., Albany C., Masterson T.A. (2017). Testicular cancer: The usage of central review for pathology diagnosis of orchiectomy specimens. Urol. Oncol..

